# Comparative genomics and tadpole intestinal metabarcoding suggest a potential cyanobacteria-amphibian trophic link in hot-spring ecosystems

**DOI:** 10.3389/fmicb.2026.1871509

**Published:** 2026-09-09

**Authors:** Tetsushi Komoto, Linh Thi Thuy Cao, Haruki Takayanagi, Hinayo Ogino, Haruto Koyama, Satoru Watanabe, Junpei Nomura, Ryuichi Hirota, Takeshi Igawa

**Affiliations:** 1 Amphibian Research Center, Hiroshima University, Hiroshima, Japan; 2 Institute of Microalgal Technology, Hiroshima, Japan; 3 Program of Biotechnology, Graduate School of Integrated Sciences for Life, Hiroshima University, Hiroshima, Japan; 4 Yuzawa Geopark Promotion Council, Akita, Japan; 5 Program of Biomedical Science, Graduate School of Integrated Sciences for Life, Hiroshima University, Hiroshima, Japan; 6 Department of Bioscience, Faculty of Life Sciences, Tokyo University of Agriculture, Setagaya, Japan; 7 Seto Inland Sea Carbon-Neutral Research Center, Hiroshima University, Hiroshima, Japan; 8 Program of Basic Biology, Graduate School of Integrated Sciences for Life, Hiroshima University, Hiroshima, Japan

**Keywords:** adaptive evolution, ecological association, hot-spring, microbiome, primary production

## Abstract

Hot-springs are accessible yet extreme environments for studying thermophilic microbial communities and their ecological interactions. Here, we isolated and characterized two thermophilic filamentous *Leptolyngbya*-related cyanobacterial strains, Akita and Seranma, from geothermal hot springs in Japan. The two strains differed in morphology and pigmentation: *L.* sp. Akita formed linear blue-green filaments, whereas *L.* sp. Seranma formed loosely coiled brown filaments. Whole-genome sequencing and comparative genomic analyses revealed strain- and strain-group-specific gene enrichment patterns related to nitrogen metabolism, metal binding, mobile genetic elements, and the photosynthetic apparatus, as well as candidate genes putatively associated with thermal tolerance. *L.* sp. Seranma also showed light-dependent pigmentation changes and retained an *rfpABC* gene cluster, indicating light-responsive phenotypic and genomic variation. Intestinal 16S rRNA gene metabarcoding of sympatric *Buergeria* tadpoles detected *Leptolyngbya*-assigned reads, particularly in *B. japonica* from Seranma hot-spring. These reads showed a close correspondence to *L.* sp. Seranma, suggesting possible exposure to, or recent ingestion of, *L.* sp. Seranma-related cyanobacteria. Together, these findings provide genomic and metabarcoding evidence for a potential trophic association between thermophilic cyanobacteria and amphibian larvae in hot-spring ecosystems.

## Introduction

Cyanobacteria are essential microorganisms that act as primary producers in aquatic ecosystems and play a pivotal role in sustaining the food web dynamics. In extreme environments, where nutrient and biomass availability is constrained, environmental adaptation and establishment of cyanobacteria are critical events that influence the local ecosystems (Etesami, 2025; Klatt et al., 2011; Xu et al., 2025). Temperature is one of the most fundamental environmental factors to which all organisms, including cyanobacteria, must adapt during evolution and the expansion of their distribution ranges (Coello-Camba and Agustí, 2024; Garcia-Pichel et al., 2013; Kees et al., 2022; Paerl and Huisman, 2009).

Hot-spring ecosystems represent naturally high-temperature aquatic environments and provide valuable systems for studying thermophilic microorganisms. In these environments, cyanobacteria can maintain primary productivity and thereby support higher trophic levels in otherwise nutrient-limited environments. Furthermore, they are increasingly recognized as producers of bioactive nutrient compounds, including proteins, carbohydrates, essential fatty acids, vitamins, minerals, and other functional bioactive compounds, which have potential applications in food or supplements (Vaz et al., 2016). Additionally, the metabolic pathways of thermophilic cyanobacteria have evolved to enable the synthesis of unique compounds that can be harnessed for biotechnological applications, thereby broadening their significance beyond their ecological contributions (Rasul et al., 2024). Collectively, these properties highlight the ecological importance of cyanobacteria not only as primary producers but also as key contributors to energy flow and ecosystem stability in extreme environments (Pandey et al., 2025).

In a unique ecosystem adapted to high-temperature environments, we recently discovered two analogous cases of coexistence between cyanobacteria and anuran amphibians of the genus *Buergeria* in natural hot-springs in Japan. One of the *Buergeria* species, *B. japonica*, known as the “hot-spring frog,” has tadpoles that thrive at the highest recorded water temperature (46.1 °C) in natural hot-springs on Kuchinoshima Island, Kagoshima Prefecture (Komaki et al., 2016). Another species, *B. buergeri*, has also been reported to inhabit a river where a natural hot-spring occurs in Kawara-no-yukko, Yuzawa City, Akita Prefecture (Takayanagi, 2019). Interestingly, both populations of *Buergeria* species coexist with microbial mats consisting of cyanobacteria and utilize them as food.

Amphibians are crucial vertebrates that serve as omnivores during the tadpole stage and as consumers after metamorphosis within the trophic level. Moreover, nutrient regeneration stemming from both herbivorous and carnivorous feeding activities of tadpoles can enhance the productivity of benthic algae, phytoplankton, and herbivorous benthic chironomid larvae (Iwai et al., 2012). Consequently, both local ecosystems, initiated by cyanobacteria and further structured through amphibian–microbial interactions, provide valuable models for understanding ecological and evolutionary processes under extreme environmental conditions.

In this study, we isolated two thermophilic cyanobacterial strains from hot-spring microbial mats in Japan and characterized their morphology, genomes, and functional genomic features. We also performed intestinal 16S rRNA gene metabarcoding of sympatric *Buergeria* tadpoles to evaluate whether *Leptolyngbya*-related cyanobacteria are detected in tadpole intestines. Together, these analyses provide insights into thermophilic cyanobacterial diversity and a potential amphibian–cyanobacteria dietary association in high-temperature aquatic ecosystems.

## Materials and methods

### Sample collection, isolation, and growth conditions

Cyanobacteria were isolated from microbial mats collected at two geothermal hot-spring sites in Japan: one from “Kawara-no-yukko” (hot-spring in the Yakunaigawa River), Akinomiya, Akita Pref., in August 2022 and the other from Seranma hot-spring, Kuchinoshima Island, Kagoshima Pref. in June 2023 (Supplementary Figure 1). At both sites, microbial mats were found in small water streams originating from the hot-springs (43.8 °C at “Kawara-no-yukko” and over 50 °C at Seranma hot-spring) and eaten by tadpoles of frogs which belong to the genus *Buergeria* (*B. buergeri* in “Kawara-no-yukko” and *B. japonica* in Seranma hot-spring). The sampling strategy differed between Kawara-no-yukko and Seranma hot-spring because the spatial distributions of tadpoles and microbial mats differed between the two sites. At Kawara-no-yukko, the Yakunaigawa River has continuous water flow and a relatively broad stream area, and tadpoles of *B. buergeri* were not strongly associated with sites where microbial mats were well-developed. We therefore sampled tadpoles across a relatively wide range of accessible thermal habitats. In contrast, near Seranma hot-spring, water flow was limited, and both tadpoles of *B. japonica* and microbial mats were restricted to small, pool-like habitats where they tended to co-occur. Sampling at Seranma hot-spring was therefore conducted in representative habitats where tadpoles and microbial mats were present. Microbial mats were collected using sterile forceps and spoons and transferred into 50 mL tubes containing local hot-spring water. In the laboratory, mat fragments were rinsed with sterile distilled water and inoculated into BG11 liquid medium. Cultures were incubated at 34 °C under continuous illumination to enrich phototrophic cyanobacteria, followed by repeated subculturing. Aliquots were then spread onto BG11 agar plates and incubated at 40 °C to isolate thermotolerant cyanobacterial colonies. Colonies with consistent filamentous morphologies were transferred to liquid BG11 medium for further purification. Through this procedure, two purified cyanobacterial cultures were obtained from geographically distinct hot-spring sites and used for further phenotypic and genomic characterization.

### Light-dependent pigmentation experiments and genomic analysis of *rfp* gene regions

Light-dependent pigmentation experiments were performed using a multi-cultivator system (MC-1000; Photon Systems Instruments, Czech Republic) equipped with programmable light-emitting diode (LED) light modules. Cyanobacterial cultures were incubated at 40 °C under continuous illumination of white, red, or green LED light, each set to a photosynthetic photon flux density (PPFD) of 60 μmol photons m^–2^ s^–1^. The peak emission wavelengths of the red and green LEDs were approximately 615 and 530 nm, respectively. Absorption spectra of intact cells were measured using a UV–visible spectrophotometer and normalized to OD680. To assess whether the spectral responses are associated with the genomic organization of *rfp* genes, we examined the genomic regions surrounding *rfpA*, *rfpB*, and *rfpC* in two novel strains and *L.* sp. JSC-1. In *L.* sp. JSC-1, a genomic region encompassing *rfpA*, *rfpB*, and *rfpC* (pgaptmp_006202, pgaptmp_006201, and pgaptmp_006203, respectively) was extracted from NZ_KL662192.1 (positions 1,090,578–1,121,582).

### Genomic DNA extraction and sequencing of two cyanobacterial isolates

Genomic DNA from two purified cyanobacterial cultures was extracted for whole-genome sequencing using the DNeasy PowerBiofilm Kit (Qiagen, Hilden, Germany), and libraries for the Oxford Nanopore Technology (ONT) sequencer were constructed using a ligation library preparation kit (SQK-LSK114, ONT). All procedures were performed according to the manufacturer’s instructions, and the quality and molecular weight of genomic DNA were measured using a Qubit fluorometric quantification system (Thermo Fisher Scientific, Inc.). Sequencing was conducted using a MinION sequencer (MinION Mk1B, ONT) and a Flongle flow cell (FLO-FLG114, ONT). The raw sequence data were base-called using a super-accuracy model with duplex base calling, utilizing Dorado (version 0.9.5) and duplex tools (version 0.3.3).

### Genome assembly, assessment of completeness, gene prediction, and annotation

Sequenced reads with a q-score of >10 were assembled using Flye (v2.9.4-b1799) (Kolmogorov et al., 2019) with the “–nano-hq” parameter. Genome completeness was assessed using the Benchmarking Universal Single-Copy Orthologs [BUSCO (version: 5.7.0)] gene set (cyanobacteria_odb10) (Manni et al., 2021) and CheckM2 (version: 1.1.0) (Chklovski et al., 2023). These two assembled genome sequences and published genomic FASTA files of other Leptolyngbya-related strains (Supplementary Table 1) were used for gene prediction and annotation using the prokaryotic gene annotation pipeline PGAP (version: 2024-07-18. build7555) (Tatusova et al., 2016). Functional domains in each gene were estimated using InterProScan (version 5.59-91.0) (Jones et al., 2014), and Pfam IDs obtained from InterProScan were translated to GO terms using pfam2go in ragp (version 0.3.5.9000) (Dragićević et al., 2020).

The *rfpA*, *rfpB*, and *rfpC* genes in *L.* sp. Seranma and *L.* sp. JSC-1 (Taxonomy ID: 1487953, RefSeq ID: GCF_000733415.1) were identified based on PGAP annotations. Genomic regions corresponding to the *rfpABC* cluster and adjacent genes were extracted from the genomes of *L.* sp. Seranma and *L.* sp. JSC-1. To assess gene content, order, and orientation within the *rfpABC* loci, visualization of gene cluster organization and calculation of sequence similarity were conducted using Geneious (version: 9.1.8) (Kearse et al., 2012).

### Molecular phylogenetic and genome-wide similarity analyses

First, to infer the phylogenetic placement of our isolated strains in the phylum Cyanobacteriota, we analyzed the 16S rRNA gene sequences of strains Akita and Seranma together with all reference 16S rRNA gene sequences included in Cydrasil (Roush et al., 2021). The combined sequence dataset was realigned using MAFFT (version 7.525, parameters: –auto) (Katoh and Standley, 2013), and a phylogenetic tree was reconstructed using IQ-TREE (version 2.4.0, parameters: -m MFP -B 1000 -nm 5000) (Minh et al., 2020). To further evaluate genome-wide relatedness, we calculated average nucleotide identity (ANI) and digital DNA–DNA hybridization (dDDH) values. RefSeq genome assemblies assigned to Cyanobacteriota were obtained from NCBI using NCBI Datasets (O’Leary et al., 2024). In total, 2,633 genome assemblies were retrieved and used as the reference dataset. ANI values and aligned fractions were calculated using skani (Shaw and Yu, 2023) by comparing the longest contig of each query against the list of RefSeq genome FASTA files. dDDH values between the query strains and closely related reference genomes were estimated using the Type Strain Genome Server (TYGS) (Meier-Kolthoff and Göker, 2019). Based on the Cydrasil placement, genome-wide similarity screening, sequence similarity of 16S rRNA genes and 16S–23S ITS regions, filamentous morphology and thermophilic ecology, we then conducted genus-level phylogenetic analyses using 16S rRNA gene sequences and Unicore (universal and core) genes (single-copy core genes common to most members of a clade) of *Leptolyngbya*-related strains. In the 16S rRNA gene analyses, to exclude truncated copies of 16S rRNA genes, we selected copies 1,400 bp or more in length in each strain. Because no 16S rRNA gene sequence of 1,400 bp or more was found in *L.* sp. JSC-1, a sequence deposited in the NCBI database (accession ID: FJ788926) was used. After multiple alignments of 16S rRNA gene sequences using MAFFT (parameters: –auto), a phylogenetic tree was constructed using IQ-TREE (parameters: -m MFP -B 1000). In the analysis of the Unicore gene set, single-copy genes present in more than 80% of the *Leptolyngbya*-related strains were extracted. Using the pipeline included in the Unicore package (Kim et al., 2025), this gene set was concatenated, aligned, and used for phylogenetic tree construction. These phylogenetic trees with bootstrap values were visualized as unrooted trees using iTOL^1^ (Letunic and Bork, 2024). Although a broader range of cyanobacterial taxa was initially included, this resulted in unstable phylogenetic tree topologies, likely due to long-branch attraction (data not shown). The dataset was therefore restricted to the taxa described above to ensure a more reliable phylogenetic inference.

### Synteny identification and comparative genome analysis using predicted gene models

We constructed synteny plots between *L.* sp. Akita and *L.* sp. Seranma, and their phylogenetically closest strains using ReCCO (version: 1.0-beta, download link of script^2^), and the plots were visualized using DiGAlign (version: 2.0, web server^3^) (Nishimura et al., 2024) with default parameters and small modifications for proper execution.

For comparative genomic analyses, reciprocal BLASTP (version 2.15.0) (Altschul et al., 1990) searches were performed between the predicted genes of *L.* sp. Akita, *L.* sp. Seranma, and *L.* sp. JSC-1 and predicted genes of each *Leptolyngbya*-related strain. *L.* sp. JSC-1 was included as a reference strain because it is a well-characterized thermophilic *Leptolyngbya*-related cyanobacterium isolated from a hot-spring microbial mat and provides an ecologically relevant comparison for the isolated hot-spring strains. We classified the genes of *L.* sp. Akita, *L.* sp. Seranma, and *L.* sp. JSC-1 into three categories: reciprocal best hits (RBHs), non-reciprocal best hits (BHs), and no homologous hits (NHs). We used e-value ≤ 1.0e-10 in these BLASTP searches. After classifying genes into these three categories, state matrices were formed from the category vectors, and hierarchical clustering based on Gower’s distance and Ward’s method was performed. To determine the dendrogram cut thresholds, we plotted the cut height against the resulting number of clusters and selected thresholds at points where the decrease in cluster number became gradual with increasing cut height. To obtain significantly enriched Gene Ontology (GO) terms in each gene cluster, genes were divided into clusters using appropriate thresholds from the results of hierarchical clustering, and Fisher’s exact probability tests with multiplicity adjustment using the Benjamini-Hochberg method were performed for each gene cluster.

### Identification of candidate genes associated with hot-spring-derived strains

We compared gene repertoires between hot-spring-derived strains and phylogenetically related strains from non-thermal environments based on the topology of the Unicore phylogenetic tree. To identify candidate genes potentially associated with thermal adaptation in hot-spring-derived strains, we searched for two categories of genes. First, *L.* sp. Akita-specific candidate genes were defined as genes that were present in *L.* sp. Akita but absent from phylogenetically related comparison strains from non-thermal environments. Second, *L.* sp. Seranma– *L.* sp. JSC-1– *L.* sp. O-77 lineage-specific candidate genes were defined as genes that were retained as reciprocal best hits (RBHs) among *L.* sp. Seranma, *L.* sp. JSC-1, and *L.* sp. O-77, but absent from the corresponding phylogenetically related comparison strains from non-thermal environments. For the *L.* sp. Akita-related lineage, the comparison set included *L.* boryana, *L.* sp. GGD, *L.* sp. UWPOB_LEPTO1, *L.* sp. FACHB-161, *L.* sp. FACHB-238, *L.* sp. FACHB-239, *L.* sp. FACHB-402, *L.* sp. FACHB-17, and *L.* sp. NIES-2104. For the *L.* sp. Seranma–*L.* sp. JSC-1–*L.* sp. O-77-related lineage, the comparison set included *L. ohadii*, *L.* sp. FACHB-711, *L.* sp. FACHB-8, and *L.* sp. FACHB-16. Genes shared between the *L.* sp. Akita-related and *L.* sp. Seranma–*L.* sp. JSC-1–*L.* sp. O-77-related hot-spring-derived lineages were also identified by comparing the two candidate gene sets. Among the candidate genes identified by this procedure, annotations potentially associated with thermal tolerance were summarized.

### 16 S rRNA gene metabarcoding analysis of tadpole intestinal microbiota

To confirm the ecological significance of cyanobacteria in extreme environments, we verified tadpole feeding by 16S rRNA gene metabarcoding of tadpole intestinal microbiota. We obtained tadpoles of the Japanese bell-ring frog, *B. buergeri* from “Kawara-no-yukko” in August 2022 and tadpoles of the Ryukyu bell-ring frog, *B. japonica* from Seranma hot-spring in May 2022. For *B. buergeri*, three tadpoles were collected from each of three water temperatures (26 °C, 28 °C, and 37 °C), whereas nine *B. japonica* tadpoles were collected from a single water temperature of approximately 40 °C. These tadpoles were preserved in 70% ethanol after deep anesthetization using MS-222 and kept in a −30 °C freezer until DNA extraction. All animal experiments were approved by the Hiroshima University Animal Research Committee (approval number: G14-2). Total DNA, including intestinal microbial metagenomic DNA and tadpole DNA, was extracted from the tadpole intestine using DNAs-ici!-F (Rizo Inc., Tsukuba, Japan) according to the manufacturer’s instructions and dissolved in 50 μL of nuclease-free water. We amplified the full-length 16S rRNA gene using 27F (5’-AGAGTTTGATCCTGGCTCAG-3’) and 1492R (5’-TACGGYTACCTTGTTACGACTT-3’). Polymerase chain reaction (PCR) amplification was conducted in a 25 μL reaction with 0.5 U KOD FX Neo (Toyobo Co., Ltd. Osaka, Japan), 1 × PCR Buffer for KOD FX Neo, 0.4 mM each dNTP, 0.2 μM of each primer and 1 μL of total DNA template, using the following cycling conditions: initial denaturation at 94°C for 3 min, followed by 35 cycles of denaturation at 98°C for 10 s, annealing at 55°C for 30 s, and elongation at 68°C for 2 min. The remaining primers were then removed from the PCR products by polyethylene glycol precipitation and dissolved in 5 μL of nuclease-free water. After measuring the concentration using a Qubit Fluorometer and Qubit dsDNA HS Assay Kit (Thermo Fisher Scientific), each precipitated sample was used for barcoded library construction for Nanopore sequencing following the native barcoding protocol (SQK-LSK109 with EXP-NBD104 and EXP-NBD114, NBE_9065_v109_revAP_14Aug2019). Following the manufacturer’s instructions, sequencing was conducted using a MinION sequencer (MinION Mk1B, ONT) and Flongle flow cell (FLO-FLG001, ONT). MinKNOW (version: 22.03.6) was used for basecalling and demultiplexing with super-accurate basecalling model, and the resultant FASTQ files of each sample were analyzed using the EPI2ME Labs platform (version: 5.2.3) with the wf-metagenomics Nextflow workflow (version: 2.13.0) and pre-built “core-nt” Kraken2/Bracken database (downloaded 4/27/2025^4^). Differential abundance analysis was conducted using the ALDEx2 (version 1.36.0) (Fernandes et al., 2013) package, which outputs multiple statistical tests, including Welch’s *t*-test, Wilcoxon rank-sum test, Kruskal-Wallis test, and generalized linear model (GLM) with significance assessed by the Wald test, based on centered log-ratio-transformed abundances. The *p*-values were adjusted using the Benjamini-Hochberg method.

## Results

### Morphological evaluation and whole-genome sequencing of the two cyanobacterial isolates

**FIGURE 1:**
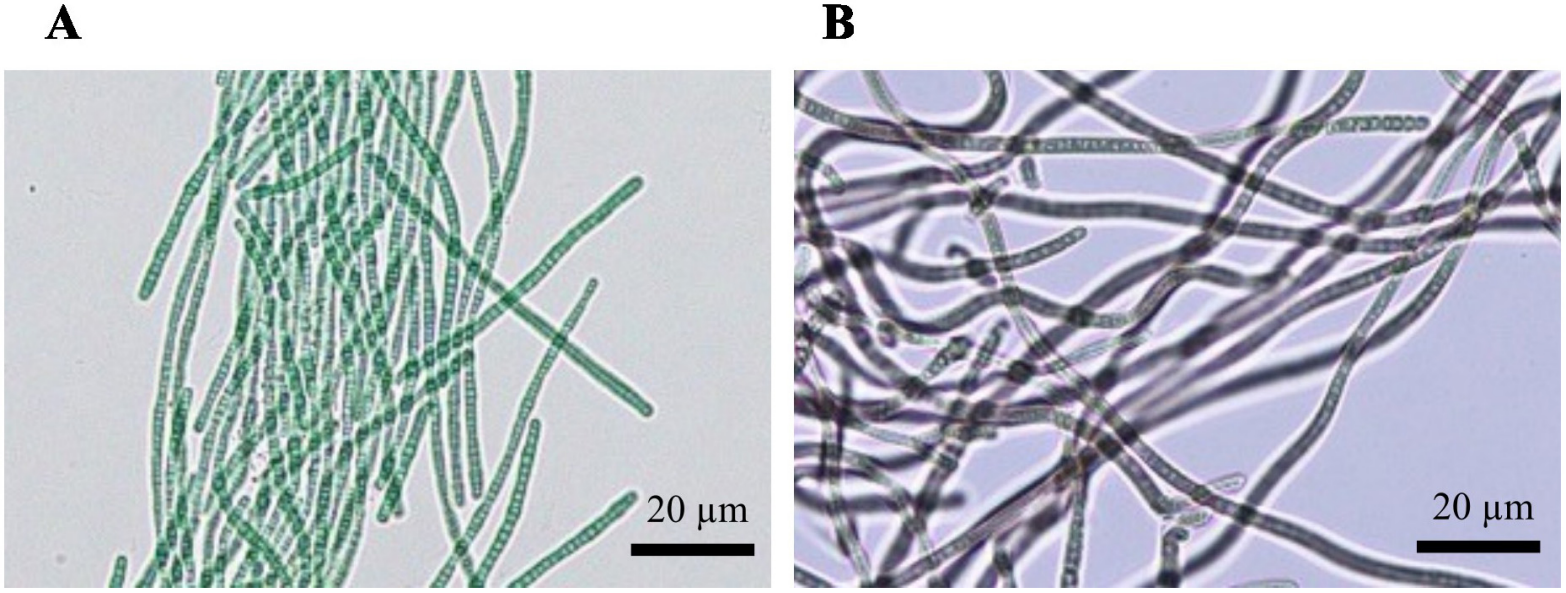
Photographs of the novel isolated strains. Microphotographs of **(A)** *L.* sp. Akita, and **(B)** *L.* sp. Seranma.

After repeated subcultures of microbial mats collected from hot-springs in Kawara-no-yukko and Seranma, fine green and brown cyanobacterial strains were obtained. Both strains exhibited filamentous and non-heterocystous structures under a light microscope (Figures 1A, B). Based on their morphology, hot-spring habitat, molecular phylogenetic relationships, and genome-wide similarity analyses described below, the two isolates were regarded as *Leptolyngbya*-affiliated cyanobacteria and hereafter referred to as *L.* sp. Akita and *L.* sp. Seranma, respectively, after their collection sites. *L.* sp. Akita exhibited a relatively linear filamentous structure, whereas *L.* sp. Seranma exhibited a loosely coiled filamentous structure.

We obtained 405,522,732 and 933,140,049 bp of raw sequence data for *L.* sp. Akita and *L.* sp. Seranma (Table 1). The resultant genome assemblies based on these data consisted of three circular and one linear contig for *L.* sp. Akita (6,829,225 bp in total) and two circular and two linear contigs for *L.* sp. Seranma (8,049,769 bp in total) (Table 1). The BUSCO completeness scores of these genomes were 95.5% and 99.6%, with corresponding missing rates of 1.4% and 0.3%, respectively. CheckM2 estimated genome completeness values of 99.5% and 100.0%, with contamination levels of 3.1% and 2.49%, respectively.

**TABLE 1:** Metrics of the draft genomes of strains Akita and Seranma with Benchmarking Universal Single-Copy Orthologs (BUSCO) completeness.

Strain	Akita	Seranma
Total number of reads	98,144	163,820
Total length of reads (bp)	405,522,732	933,140,049
N50 of reads (bp)	17,945	6,502
>Q20 (%)	65.18	81.28
>Q30 (%)	35.04	67.18
Total number of contigs	4	4
Total assembly size (bp)	6,829,225	8,049,769
GC content (%)	46.75	50.77
Assembly size and state of contigs (bp)	contig_1: LC, 277,981	contig_1: LC, 1,313,761
	contig_3: CC, 6,300,885	contig_2: CC, 6,476,496
	contig_4: CC, 10,498	contig_3: LC, 226,323
	contig_5: CC, 239,861	contig_4: CC, 33,189
BUSCO completeness (%, cyanobacteria_odb10, 773 genes from 141 genomes)	Complete: 95.5 (single-copy: 95.2, duplicated: 0.3)	Complete: 99.6 (single-copy: 98.6, duplicated: 1.0)
	Fragmented: 3.1	Fragmented: 0.1
	Missing: 1.4	Missing: 0.3
CheckM2 (%)	Completeness: 99.5	Completeness: 100.0
	Contamination: 3.1	Contamination: 2.49
Total number of estimated genes from PGAP (genes)	6,307	6,752

Linear and circular contigs are indicated as LC and CC, respectively.

### Light-dependent absorption spectral changes and conservation of the *rfpABC* gene cluster

*L.* sp. Seranma showed light-dependent pigmentation changes under the tested conditions, appearing brown under white light and green under red light (Supplementary Figure 2), whereas *L.* sp. Akita showed no obvious pigmentation change. In *L.* sp. Akita, the absorption spectra remained largely unchanged across the different light conditions (Supplementary Figure 2). In contrast, *L.* sp. Seranma exhibited light-dependent changes in its absorption spectra with shifts in the spectral distribution among cells grown under white, red, and green light conditions (Supplementary Figure 2).

**FIGURE 2:**
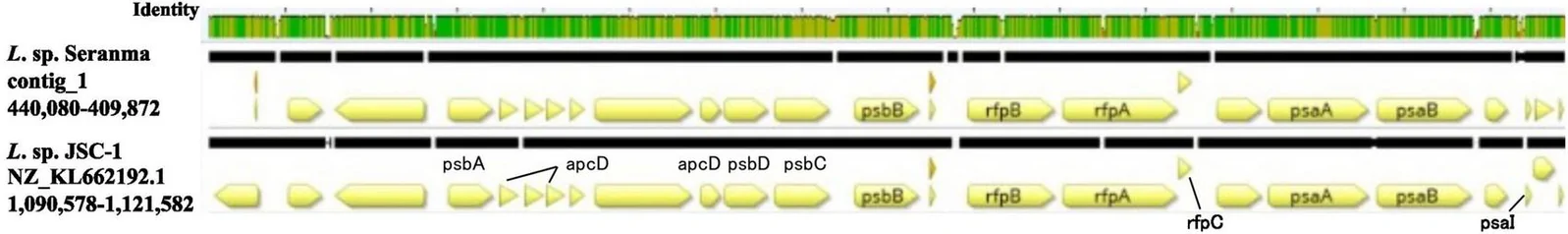
Sequence alignment of the flanking regions was visualized using Geneious, with identity values displayed according to Geneious. Gene names were assigned using PGAP. Only genes shared between *L.* sp. Seranma and *L.* sp. JSC-1 are shown, and product names are omitted for simplicity.

In addition, previous studies demonstrated that *rfpA*, *rfpB*, and *rfpC* function as master regulatory elements controlling far-red light photoacclimation (FaRLiP) and are encoded within a conserved gene cluster (Gan et al., 2014). Consistent with this, the genomic organization of the rfpABC cluster was highly conserved between *L.* sp. Seranma and *L.* sp. JSC-1, including gene order, orientation, and surrounding synteny (Figure 2). In contrast, no corresponding gene cluster was identified in *L.* sp. Akita.

### Phylogenetic relationships in cyanobacteria and *Leptolyngbya*-related strains

**FIGURE 3:**
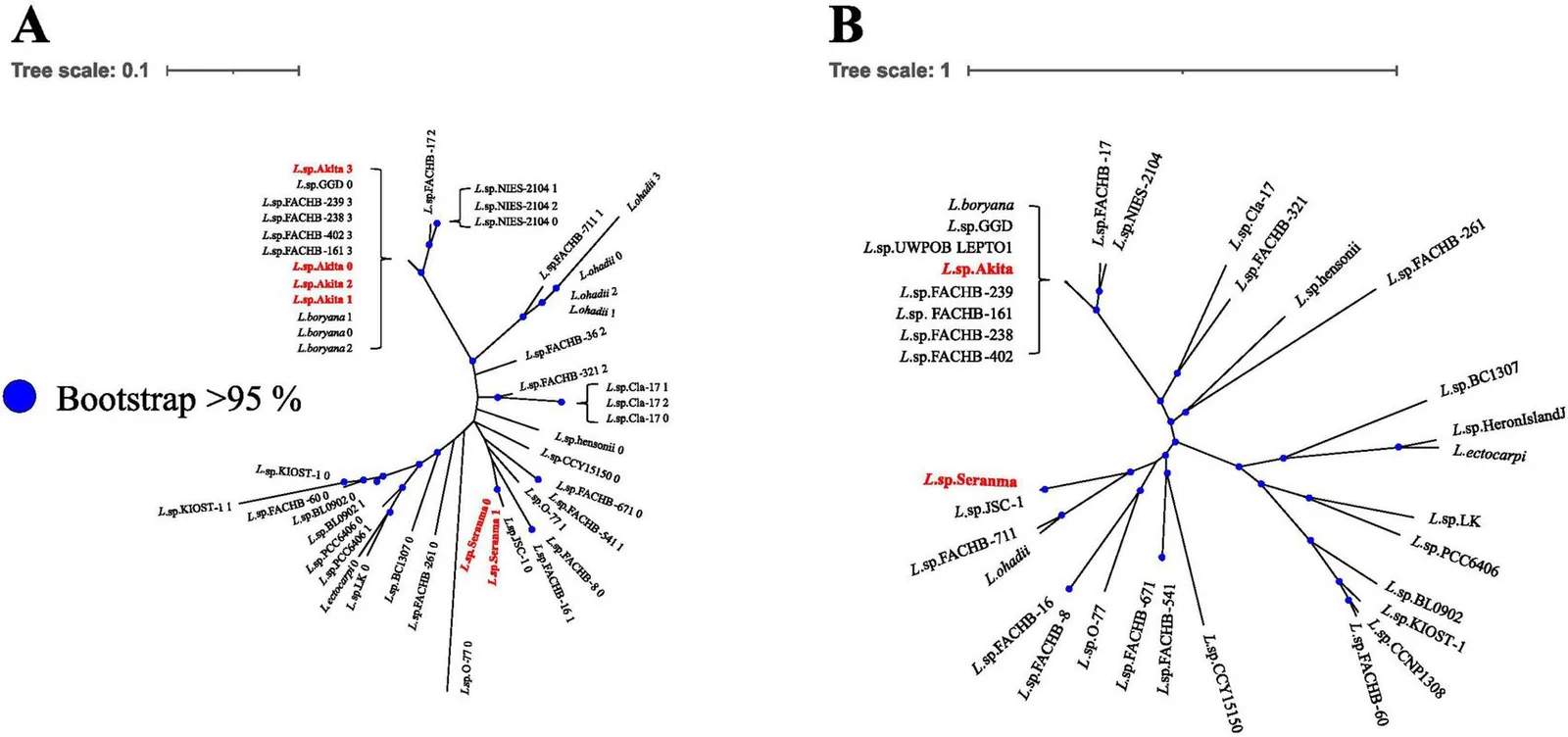
Unrooted phylogenetic trees highlighting internal nodes with bootstrap values > 95 % in *Leptolyngbya*-related strains. **(A)** 16S rRNA genes and **(B)** Unicore gene set.

The Cydrasil-based 16S rRNA gene phylogeny placed both isolates in a branch mainly composed of *Leptolyngbya*-related strains (Supplementary Figure 3 and Supplementary Table 2). RefSeq-wide ANI screening was then performed to evaluate genome-wide similarity to publicly available cyanobacterial genomes. The genome of strain Akita showed the highest ANI values to *L. boryana*-related genomes, whereas the genome of strain Seranma showed the highest ANI to *Leptolyngbya* sp. NK1-12 (Supplementary Table 3). In the TYGS-based dDDH analysis, *Planktothrix agardhii* NIES 204 was returned as the closest type-strain match for strain Akita, with a formula d4 value of 29.7%, whereas *Iningainema tapete* BLCC-T55 was returned as the closest type-strain match for strain Seranma, with a formula d4 value of 32.9% (Supplementary Tables 4, 5). We next performed more focused phylogenetic analyses using 16S rRNA genes and the Unicore gene set for the two isolates and selected *Leptolyngbya*-related strains (except for the strains with extremely low BUSCO completeness). Based on the PGAP annotation, 75 copies of 16S rRNA genes were identified in the *Leptolyngbya*-related strain genomes, and 48 of these (including a manually added sequence of *L.* sp. JSC-1) were extracted as valid 16S rRNA genes and used for phylogenetic analysis. In the phylogenetic tree of 16S rRNA genes, *L.* sp. Akita was placed in a cluster containing *L. boryana*, *L.* sp. FACHB-161, *L.* sp. FACHB-238, *L.* sp. FACHB-239, *L.* sp. FACHB-402, and *L.* sp. GGD, whereas all copies of *L.* sp. Seranma were placed in clusters containing *L.* sp. JSC-1 (Figure 3A). However, the evolutionary relationships between the strains within the clusters were unclear.

Using the Unicore pipeline, 408 proteins were selected as the Unicore gene set, and multiple sequence alignment resulted in 100,760 amino acids (including gaps). The phylogenetic tree of the Unicore gene set showed a clustering pattern similar to that of 16S rRNA genes and clear evolutionary relationships among the strains within the clusters. In the Unicore tree, *L.* sp. GGD was the strain closest to *L.* sp. Akita and *L.* sp. JSC-1 was the strain closest to *L.* sp. Seranma (Figure 3B).

### Synteny conservation patterns between *Leptolyngbya*-related strains

**FIGURE 4:**
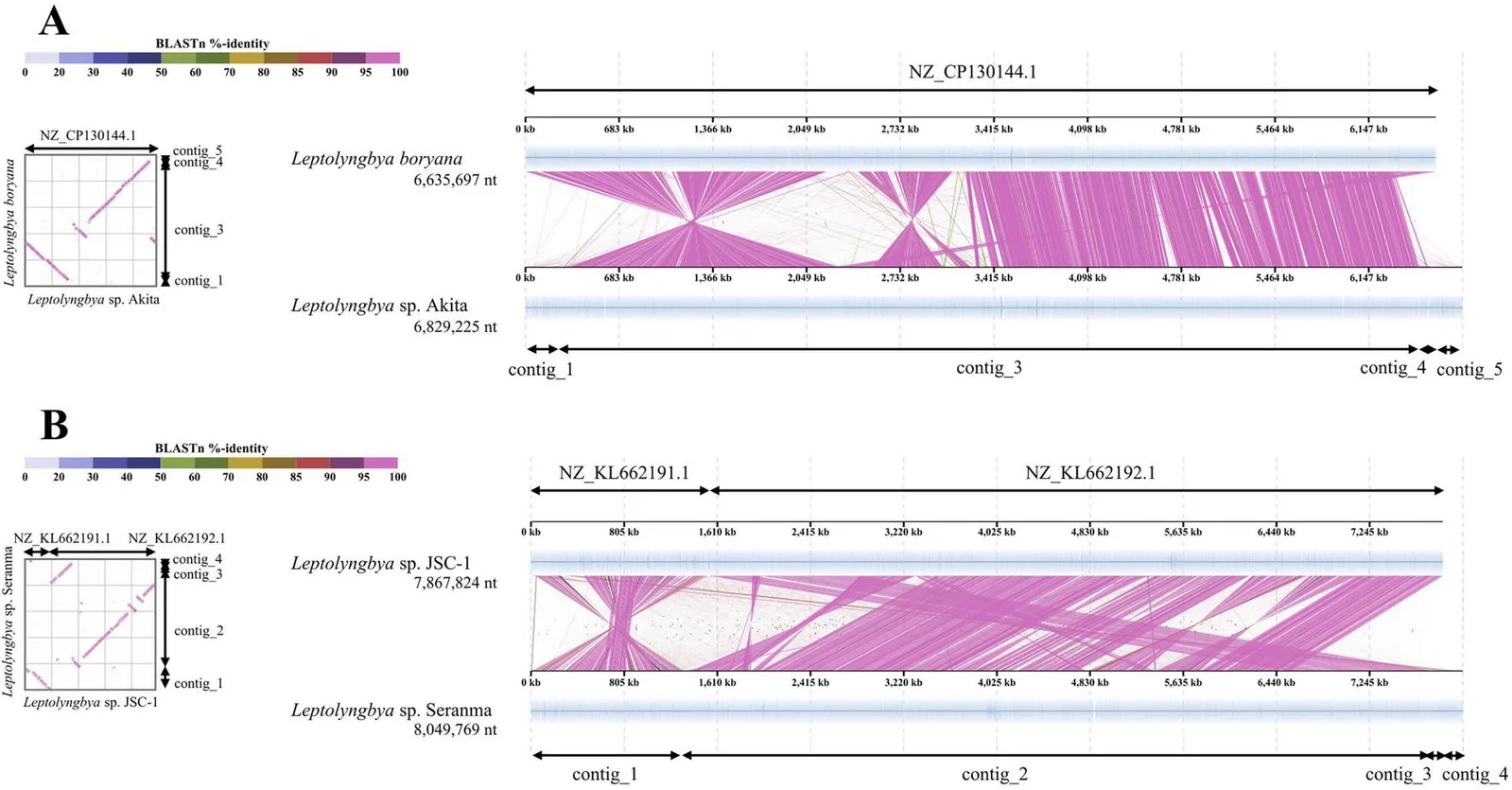
Synteny and dot plots between pairs of *Leptolyngbya*-related strains. **(A)** *L.* sp. Akita and *L. boryana*; **(B)** *L.* sp. Seranma and *L.* sp. JSC-1.

Based on the phylogenetic analyses, synteny analyses were performed between each isolate and its closest relative: *L.* sp. Akita and *L. boryana* (Figure 4A), and *L.* sp. Seranma and *L.* sp. JSC-1 (Figure 4B). The synteny plot (Figure 4A) showed macro synteny between contig_3 of *L.* sp. Akita and the chromosome of *L. boryana* (NZ_CP130144.1) with 95%–100% identity and two large genomic inversions of approximately 0.7–2 Mb. Interestingly, *L. boryana* had few homologous genomic regions with contig_1 (approximately 0.3 Mb, 5’ end in Figure 4A) in *L.* sp. Akita. The synteny plot (Figure 4B) also showed macro-synteny over the entire length of contig_2 in *L.* sp. Seranma and the scaffold of *L.* sp. JSC-1 (NZ_KL662191.1) with an identity of 95%–100%, except for three genomic inversions of approximately 0.4 Mb and four genomic transpositions of approximately 40–80 kb. In addition, the other scaffold in *L.* sp. JSC-1 (NZ_KL662192.1) showed high homology with contig_1 of *L.* sp. Seranma.

### Strain- or strain-group-specific gene enrichment and functional estimation based on comparative genomic analyses

**FIGURE 5:**
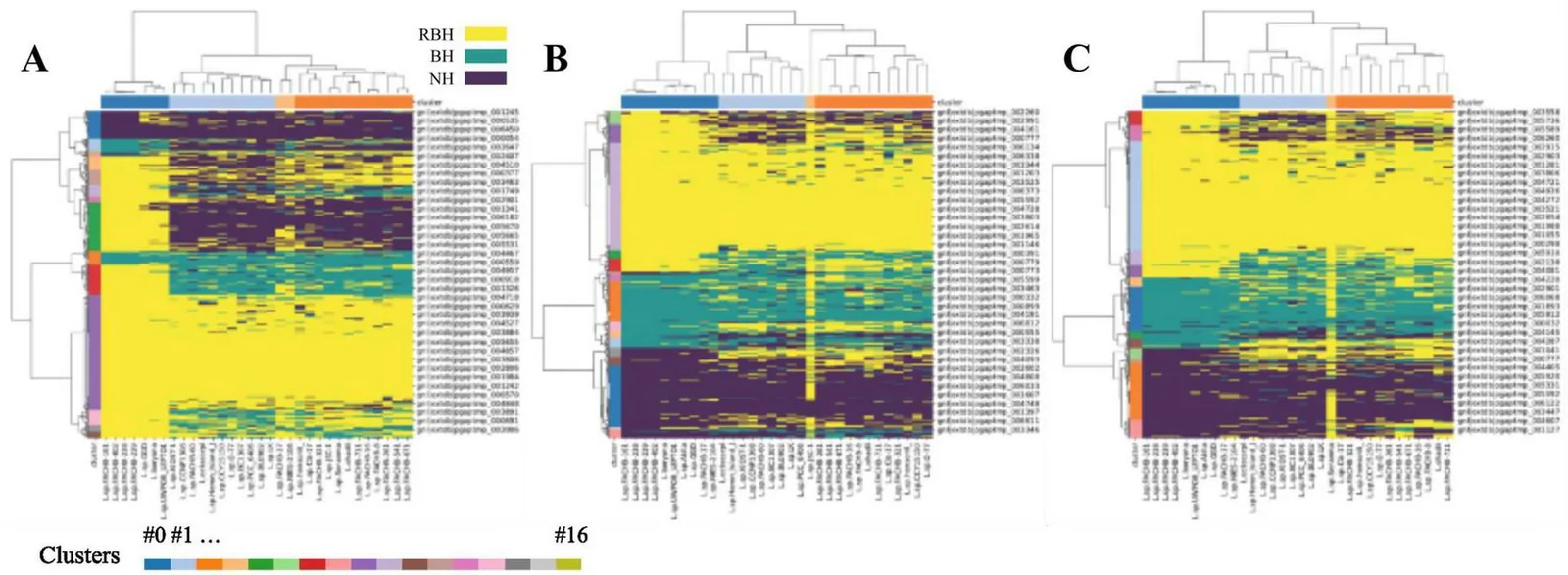
Matrices showing reciprocal BLASTP searches between genes of the target strain and genes of the other *Leptolyngbya*-related strains with dendrograms. The target strains were **(A)** *L.* sp. Akita, **(B)** *L.* sp. Seranma, and **(C)** *L.* sp. JSC-1, respectively. Genes or strains were sorted and colored according to their clusters. See Supplementary Tables 10–12 for Pfam annotations and Supplementary Tables 13–15 for enriched GO terms in each cluster.

Based on the PGAP annotation pipeline, 6,307, 6,752, and 6,354 genes were predicted in the genome sequences of *L.* sp. Akita, *L.* sp. Seranma, and *L.* sp. JSC-1 (see Supplementary Table 1 for predicted genes, including other *Leptolyngbya*-related strains). Reciprocal BLASTP searches were performed between the predicted genes of *L.* sp. Akita (Supplementary Table 6), *L.* sp. Seranma (Supplementary Table 7), or *L.* sp. JSC-1 (Supplementary Table 8) and those of each *Leptolyngbya*-related strain. Hierarchical clustering was performed using the BLASTP hit category matrices of *L.* sp. Akita, *L.* sp. Seranma, and *L.* sp. JSC-1 (Figure 5). From the dendrograms obtained by hierarchical clustering, all predicted genes of *L.* sp. Akita, *L.* sp. Seranma, and *L.* sp. JSC-1 strains were divided into 15, 13, and 12 gene clusters by selecting appropriate threshold values (Supplementary Table 9 and Supplementary Figure 4). Enrichment analyses were performed for the strain-specific gene clusters, which contained genes that specifically accumulated in the target strains and for which few homologs were found in the other strains. These clusters necessarily accumulated the highest number of NH genes, and GO terms, “nitrogen compound metabolic process” and “transition metal ion binding” were identified as significantly enriched terms [q-value (false discovery rate) < 0.05 and odds ratio > 1] in gene cluster #0 of *L.* sp. Akita (Table 2A and Supplementary Tables 10, 13). In addition, “DNA transposition” and “transposase activity” were identified as significantly enriched terms in gene cluster #0 of *L.* sp. Seranma (Table 2B and Supplementary Tables 11, 14). However, no significantly enriched GO terms were identified in gene cluster #2 of *L.* sp. JSC-1 (Supplementary Table 15). Instead, “phycobilisome” and “photosynthesis”-related GO terms were identified as significantly enriched in gene cluster #2 of *L.* sp. Seranma (Table 2C and Supplementary Tables 11, 14) and gene cluster #0 of *L.* sp. JSC-1 (Table 2D and Supplementary Tables 12, 15). The genes included in these clusters tended to be orthologs because they were only found in synteny blocks of these closely related strains (*L.* sp. Seranma and *L.* sp. JSC-1), but may not be present in other strains.

**TABLE 2:** Enriched statistically significant Gene Ontology (GO) terms.

A. GO terms in cluster #0 of *L*. sp. Akita	Odds ratio	*P*-value	*q*-value
Nitrogen compound metabolic process	3.929595	0.002765	0.024888
Transition metal ion binding	9.431028	3.14E-05	0.000848
**B. GO terms in cluster #0 of *L*. sp. Seranma**	**Odds ratio**	***P*-value**	***q*-value**
DNA transposition	3.012703	0.000855	0.007693
Transposase activity	3.012703	0.000855	0.007693
**C. GO terms in cluster #2 of *L*. sp. Seranma**	**Odds ratio**	***P*-value**	***q*-value**
ADP binding	5.244827	0.000105	0.001732
Calcium ion binding	3.337617	6.06E-08	1.79E-06
Chlorophyll binding	5.403762	0.003696	0.039071
Electron transporter, transferring electrons within the cyclic electron transport pathway of photosynthesis activity	5.67395	0.001406	0.018914
Phosphorelay sensor kinase activity	3.139509	1.35E-10	5.00E-09
Phosphorelay signal transduction system	3.215825	6.79E-14	1.01E-11
Photosynthesis	2.770283	3.02E-05	0.00056
Photosynthesis, light reaction	5.268667	2.44E-05	0.000516
Photosynthetic electron transport chain	4.863385	0.005365	0.048788
Photosynthetic electron transport in photosystem II	5.67395	0.001406	0.018914
Photosystem	5.403762	0.003696	0.039071
Phycobilisome	3.868602	3.94E-06	9.72E-05
Protein binding	3.011933	4.93E-12	2.61E-10
Protein kinase activity	2.786315	0.005871	0.048788
Protein-phycocyanobilin linkage	4.364577	0.004156	0.041006
Regulation of DNA-templated transcription	1.713048	0.003155	0.038908
Signal transduction	3.221473	5.30E-12	2.61E-10
**D. GO terms in cluster #0 of *L*. sp. JSC-1**	**Odds ratio**	***P*-value**	***q*-value**
ADP binding	5.004463	0.000666	0.01147
DNA-binding transcription factor activity	2.185412	0.001194	0.016823
Calcium ion binding	4.72987	2.06E-15	3.20E-13
Phosphorelay sensor kinase activity	2.428381	5.21E-06	0.000161
Phosphorelay signal transduction system	2.991173	3.18E-12	2.47E-10
Photosynthesis, light reaction	4.677374	0.000261	0.005782
Phycobilisome	3.011945	0.000847	0.013136
Protein binding	2.452584	1.04E-07	5.36E-06
Regulation of DNA-templated transcription	2.05933	4.35E-05	0.001124
Sequence-specific DNA binding	3.373379	0.000521	0.010101
Signal transduction	2.507906	2.89E-07	1.12E-05

### Candidate genes associated with thermal stress adaptation of hot-spring-derived strains

Using the lineage-based comparison described above, we identified 74 candidate annotations in *L.* sp. Akita and 40 candidate annotations in the *L.* sp. Seranma-*L.* sp. JSC-1-*L.* sp. O-77 lineage within the comparison set used here. Of these, three annotations were shared between *L.* sp. Akita and *L.* sp. Seranma-*L.* sp. JSC-1-*L.* sp. O-77 candidate gene sets, whereas 71 and 37 annotations were specific to *L.* sp. Akita and to *L.* sp. Seranma-*L.* sp. JSC-1-*L.* sp. O-77 lineage, respectively. The three shared annotations did not include functions with clear literature-supported links to thermal tolerance (Supplementary Table 16). Among the *L.* sp. Akita-specific annotations, several had potential links to thermal tolerance based on previous literature. These included AAA family ATPase and AAA domain-containing protein, which have been reported in relation to ATP-dependent protein remodeling and protein quality control. Other *L.* sp. Akita-specific annotations included FtsK/SpoIIIE domain-containing protein, RusA family crossover junction endodeoxyribonuclease, and UvrD-helicase domain-containing protein, which are related to cell division, recombination, or DNA repair (Tables 3A and Supplementary Table 17). In the *L.* sp. Seranma–*L.* sp. JSC-1–*L.* sp. O-77 lineage-specific candidate gene set, chaperone modulator CbpM was the only annotation summarized as potentially related to thermal tolerance based on previous literature (Table 3B and Supplementary Table 18).

**TABLE 3:** Summary of annotations potentially associated with thermal tolerance and their corresponding reference literature.

A. PGAP annotations of Akita-specific candidate genes	Gene IDs in *L*. sp. Akita	References
AAA family ATPase	pgaptmp_000094, pgaptmp_004007, pgaptmp_005586	Zolkiewski et al., 2012; Weibezahn et al., 2004; Kamata et al., 2005
FtsK/SpoIIIE domain-containing protein	pgaptmp_003217	Begg et al., 1995
RusA family crossover junction endodeoxyribonuclease	pgaptmp_003395	Mahdi et al., 1996
UvrD-helicase domain-containing protein	pgaptmp_003004	Epshtein et al., 2014
AAA domain-containing protein	pgaptmp_000088, pgaptmp_000002	Zolkiewski et al., 2012; Weibezahn et al., 2004; Kamata et al., 2005
**B. PGAP annotations of Seranma-JSC-1-O-77 lineage-specific candidate gene set**	**Gene IDs in *L*. sp. Seranma**	**References**
Chaperone modulator CbpM	pgaptmp_003624	Chenoweth et al., 2007

Annotations not listed in this table are provided in Supplementary Tables 16–18.

### Comparison of intestinal microbiota of *B. buergeri* and *B. japonica* tadpoles

**FIGURE 6:**
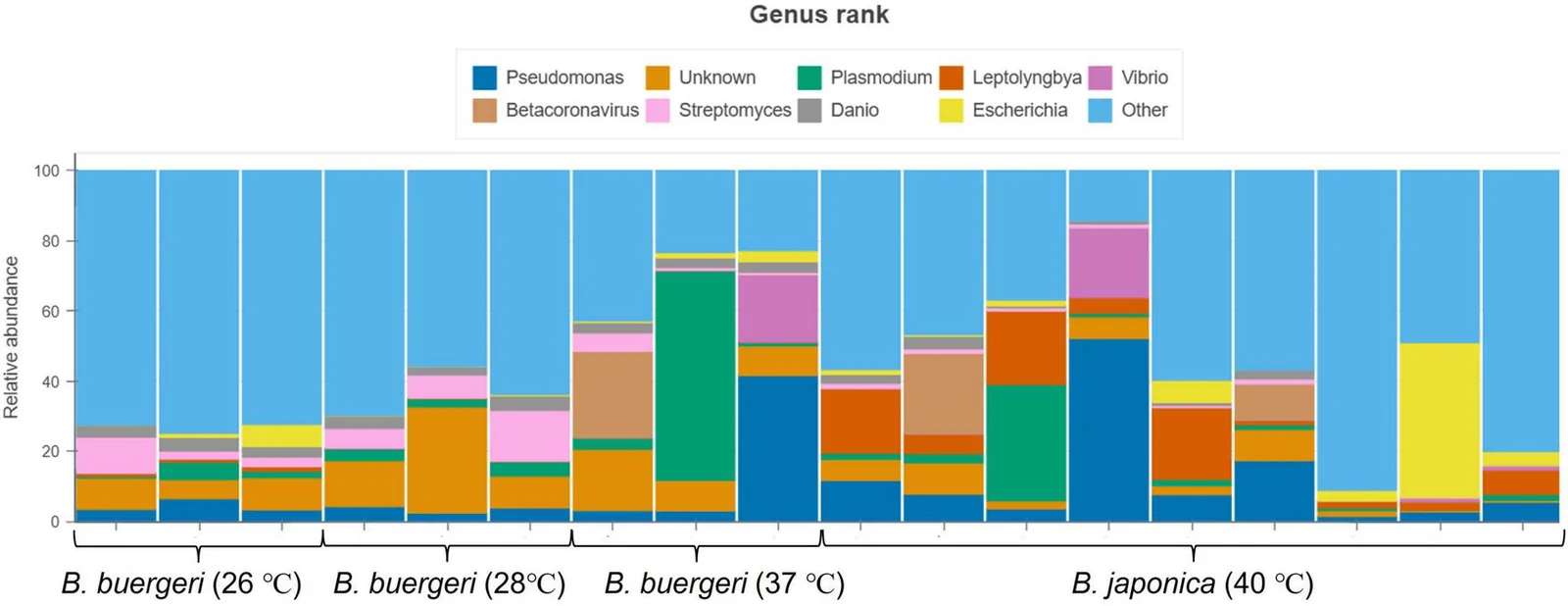
Read abundance in the intestinal metabarcoding of *B*. *buergeri* and *B*. *japonica* estimated from the Kraken2/Bracken pipeline. Barplot of the nine most abundant taxa at the genus level in all samples, where each bar represents an individual sample. Any remaining taxa were collapsed under the “Others” category to facilitate visualization. The y-axis indicates the relative abundance of each taxon as a percentage for each sample. Supplementary Tables 19–23 show the statistically significant taxa in each test.

Intestinal metabarcoding analyses of *B*. *buergeri* and *B*. *japonica* tadpoles detected 351 genera with non-zero reads using the Kraken2/Bracken pipeline. *Leptolyngbya* was detected in the intestine of *B*. *buergeri* at a maximum of 1.3%, and was not detected in all tadpoles derived from 37 °C. In contrast, *Leptolyngbya* was detected in all intestines of *B*. *japonica*, accounting for a maximum of approximately 21% (Figure 6). We then performed a statistical analysis using ALDEx2 to test which genera showed significant differences in variances or abundances among tadpole groups. The results of the Kruskal-Wallis tests showed that there was no significant difference in the abundance of any genus among *B*. *buergeri* tadpoles derived from different temperatures (26 °C, 28 °C, and 37 °C) and those of *B*. *japonica* tadpoles derived from 40 °C (*q*-value > 0.05). In contrast, the results of the GLM tests showed statistically significant differences in abundance for *Leptolyngbya* and *Limnothrix* (q-value < 0.05) (Supplementary Table 19). The results of Welch’s *t*-test showed that there was no significant difference in abundance in any comparative pairs of tadpole groups (Supplementary Tables 20–23). In contrast, the Wilcoxon tests detected several genera with significant differences among pairwise comparisons (Supplementary Tables 20–23). Among cyanobacterial taxa, *Leptolyngbya* was significantly enriched only in the comparison between all *B. buergeri* and all *B. japonica* tadpoles. We also confirmed that the 6,327 reads assigned to *Leptolyngbya* in *B. japonica* showed hits to *L.* sp. Seranma in a BLASTN search against a customized reference database to which the 16S rRNA gene sequences of *L.* sp. Seranma had been added (Supplementary Table 24).

## Discussion

### Genomic and functional differentiation of thermophilic *Leptolyngbya*-related cyanobacterial strains

We isolated two thermophilic *Leptolyngbya*-related cyanobacterial strains from hot-spring environments, both exhibiting filamentous, non-heterocystous morphology. Although the Cydrasil-based 16S rRNA gene phylogeny and RefSeq-wide ANI screening supported affiliation of the two isolates with *Leptolyngbya*-related filamentous cyanobacteria, the TYGS-based dDDH analysis did not provide informative genus- or species-level placement. The closest type-strain matches returned by TYGS were not taxonomically consistent with the *Leptolyngbya*-related lineages suggested by the other analyses. This likely reflects the limited representation of appropriate type-strain genomes for these taxonomically complex filamentous cyanobacterial lineages, rather than support for an alternative species-level assignment. Therefore, we did not attempt formal species-level assignments in this study and used *L.* sp. Akita and *L.* sp. Seranma as working taxonomic labels. Their taxonomic placement may need to be reconsidered in future revisions of *Leptolyngbya* and related filamentous cyanobacteria.

The two strains provide a useful comparison for exploring phenotypic and genomic diversification among thermophilic filamentous cyanobacteria. Genome sequencing indicated that each strain possesses a large circular contig corresponding to the putative primary chromosome, together with additional shorter contigs that may represent accessory genomic elements. The light-dependent pigmentation and *rfpABC* gene cluster analyses were included as secondary observations to characterize pigmentation-related phenotypic and genomic variation among the isolated thermophilic cyanobacteria. These analyses were motivated by visible differences in microbial mat coloration observed at the hot-spring sites. Under the tested culture conditions, *L.* sp. Seranma showed light-dependent changes in cell coloration and absorption spectra, whereas *L.* sp. Akita showed little spectral variation. However, because the red-light condition used here peaked at approximately 615 nm and did not correspond to far-red light, these spectral changes do not provide direct evidence of canonical far-red light photoacclimation (FaRLiP). At the genomic level, *L.* sp. Seranma retained the *rfpABC* gene cluster with conserved gene order and surrounding synteny similar to that of *L.* sp. JSC-1, whereas *L.* sp. Akita lacked this cluster. Therefore, the *rfpABC* gene cluster analysis should be interpreted as supplementary genomic context for light-responsive pigmentation in *L.* sp. Seranma, and functional induction of FaRLiP under far-red light conditions remains to be tested.

Genus-level phylogenetic analyses based on 16S rRNA genes and the Unicore gene set placed *L.* sp. Akita within the *L.* boryana-related lineage and *L.* sp. Seranma close to *L.* sp. JSC-1. These relationships suggest that the observed phenotypic and genomic differences have arisen among closely related filamentous cyanobacterial lineages.

Synteny analyses showed that overall chromosomal structure is largely conserved among closely related strains, whereas specific genomic regions exhibited limited homology. This pattern suggests that lineage-specific genomic regions, potentially acquired or rearranged, contribute to genome diversification within *Leptolyngbya*.

Gene composition analysis further highlighted functional differentiation between the strains. In *L.* sp. Akita, strain-specific gene clusters indicate a unique biosynthetic potential, including sequences resembling biosynthetic gene clusters (BGCs) reported in cyanobacteria (Yi et al., 2024). In contrast, the enrichment of transposon-related functions in *L.* sp. Seranma implies increased genomic plasticity (Yao et al., 2021) that may facilitate adaptation to high-temperature environments. In addition, variation in photosynthesis-related gene sets between *L.* sp. Seranma and closely related strains is consistent with differences in light acclimation strategies.

Overall, these results indicate that genome structural variation, gene content, and light-response systems collectively contribute to lineage differentiation in *Leptolyngbya*. Furthermore, gene composition analysis provides a useful framework for identifying lineage-specific functional features beyond those resolved by phylogenetic relationships alone.

### Potential roles of candidate genes in thermal adaptation of *L.* sp. Akita and the *L.* sp. Seranma–*L.* sp. JSC-1-*L.* sp. O-77 lineage

The candidate gene analysis provides hypotheses regarding genomic features that may contribute to survival in high-temperature environments. Because these candidates were inferred from comparative presence/absence patterns and PGAP annotations, they should be interpreted as putative candidates rather than direct determinants of thermotolerance. The *L.* sp. Akita-specific candidate set included annotations related to protein quality control and genome maintenance, including AAA+ ATPase-related proteins and DNA translocation, recombination, and helicase-related functions. These functions are biologically plausible under thermal stress, which can destabilize proteins and damage cellular macromolecules (Begg et al., 1995; Epshtein et al., 2014; Kamata et al., 2005; Mahdi et al., 1996; Weibezahn et al., 2004; Zolkiewski et al., 2012). In contrast, the *L.* sp. Seranma–*L.* sp. JSC-1–*L.* sp. O-77 lineage yielded fewer literature-supported candidates, with chaperone modulator CbpM representing the main annotation potentially related to protein quality-control regulation (Chenoweth et al., 2007). These patterns suggest possible lineage-specific differences in genomic features associated with thermal adaptation, but expression analyses and functional characterization will be required to test their roles in thermotolerance.

### Potential ingestion of cyanobacteria by sympatric tadpoles

Intestinal 16S rRNA gene metabarcoding detected cyanobacterial taxa, particularly *Leptolyngbya*-assigned reads, in *B. buergeri* and *B. japonica* tadpoles inhabiting hot spring environments. *Leptolyngbya*-assigned reads were more frequently detected in *B. japonica* than in *B. buergeri*, suggesting that *B. japonica* may have more frequent exposure to, or ingestion of, *Leptolyngbya*-related cyanobacteria. In addition, most *Leptolyngbya*-assigned reads from *B. japonica* showed their best BLAST hits to *L.* sp. Seranma among the reference sequences used in this study. This correspondence between the free-living isolate and intestine-derived reads supports the possibility that *B. japonica* tadpoles ingest *L.* sp. Seranma-related cyanobacteria in their natural habitat, although the 16S rRNA gene metabarcoding data do not provide strain-level proof of dietary utilization. This difference may partly reflect site-specific habitat structure and the spatial arrangement of thermal microhabitats. At Kawara-no-yukko, *B. buergeri* tadpoles occur in the Yakunaigawa River, which provides a relatively broad flowing habitat, and high-temperature, cyanobacteria-rich areas represent only part of the available tadpole habitat. In contrast, around Seranma hot-spring, available aquatic habitats for *B. japonica* tadpoles are limited to small, pool-like water bodies where high-temperature conditions, microbial mats, and tadpoles tend to overlap. Therefore, temperature may influence the amphibian–cyanobacteria association by shaping the spatial overlap between microbial mat formation and tadpole occurrence, rather than by directly inducing a trophic interaction. This spatial overlap may increase opportunities for *B. japonica* tadpoles to ingest cyanobacterial biomass, although feeding preference and cyanobacterial productivity across temperature gradients were not experimentally tested.

The heterogeneous distribution of *Leptolyngbya*-assigned reads among individuals suggests that these cyanobacteria are unlikely to represent a consistently dominant dietary component. Moreover, amplicon-based read abundance provides only an indirect estimate of dietary intake and may be affected by differential digestion, 16S rRNA gene copy number, or microbial persistence within the intestines. Therefore, the observed patterns should be interpreted as evidence of a potential dietary association rather than quantitative dietary contribution.

Although tadpoles and cyanobacteria-rich microbial mats co-occurred in hot-spring environments, the relationship between cyanobacterial ingestion and tadpole thermal tolerance remains unclear. Because feeding preference, nutritional contribution, and thermal performance were not directly tested in this study, controlled feeding experiments using isolated *Leptolyngbya*-related strains will be required to evaluate their functional role in tadpole nutrition and stress tolerance.

## Data Availability

The sequencing data for *L.* sp. Akita and *L.* sp. Seranma are available in the DDBJ Sequence Read Archive under BioProject accession number PRJDB35926.

## Footnotes

1
https://itol.embl.de/

2
https://www.genome.jp/ftp/tools/ReCCO/ReCCO_Readme.html

3
https://www.genome.jp/digalign/

4
https://benlangmead.github.io/aws-indexes/k2
