# Supplementary material for: Comparative genomics and tadpole intestinal metabarcoding suggest a potential cyanobacteria-amphibian trophic link in hot-spring ecosystems
Source: Front Microbiol. 2026 Sep 9;17:1871509. doi: 10.3389/fmicb.2026.1871509 (PMC13598712; doi:10.3389/fmicb.2026.1871509)
Supplement: Supplementary file 1 [file Data_Sheet_1.docx]

**Supplementary information**

Fig. S1 Representative photographs of sampling sites and tadpole habitats in the hot-spring environments. Arrows indicate microbial mat-rich areas, and circles indicate areas where tadpoles were observed at (A) Kawara-no-yukko and (B) Seranma hot-spring.

Fig. S2 (A) Coloration of *L*. sp. Akita and *L*. sp. Seranma grown under white, red, and green light conditions, (B) corresponding absorption spectra.

Fig. S3 A phylogenetic tree generated using 16S rRNA genes of strain Akita, strain Seranma, and curated Cydrasil database showed (A) the location of strain Akita and strain Seranma in the whole tree, (B) branch of strain Akita, and (C) branch of strain Seranma

Fig. S4

Thresholds of hierarchical clustering in (A) *L*. sp. Akita, (B) *L*. sp. Seranma, and (C) *L*. sp. JSC-1

Table S1 Metrics in draft genome of each *Leptolyngbya* strain used in this study

Table S2 Correspondence between branch names and branch IDs used in Cydrasil-based phylogenetic tree

Table S3 RefSeq-wide ANI screening of the isolated cyanobacterial genomes. ANI values and aligned fractions were calculated using skani for *L*. sp. Akita and *L*. sp. Seranma against publicly available Cyanobacteriota genome assemblies.

Table S4 TYGS-based dDDH analysis of *L*. sp. Akita

Table S5 TYGS-based dDDH analysis of *L*. sp. Seranma

Table S6 Best hit protein IDs and categories of reciprocal BLASTP search between predicted proteins of *L.* sp. Akita and those of each *Leptolyngbya* strain

Table S7 Best hit protein IDs and categories of reciprocal BLASTP search between predicted proteins of *L.* sp. Seranma and those of each *Leptolyngbya* strain

Table S8 Best hit protein IDs and categories of reciprocal BLASTP search between predicted proteins of *L.* sp. JSC-1 and those of each *Leptolyngbya* strain

Table S9 Cluster IDs of each protein in *L*. sp. Akita, *L*. sp. Seranma, and *L*. sp. JSC-1

Table S10 Results of InterProScan (Pfam) in *L*. sp. Akita

Table S11 Results of InterProScan (Pfam) in *L*. sp. Seranma

Table S12 Results of InterProScan (Pfam) in *L*. sp. JSC-1

Table S13 Significantly enriched GO terms in each gene cluster on *L.* sp. Akita

Table S14 Significantly enriched GO terms in each gene cluster on *L.* sp. Seranma

Table S15 Significantly enriched GO terms in each gene cluster on *L.* sp. JSC-1

Table S16 PGAP annotations, those mapped to genes that were RBH in hot spring-derived strains and NH in non-thermal environment-derived strains, shared between *L*. sp. Akita and *L*. sp. Seranma– *L*. sp. JSC-1- *L*. sp. O-77 lineage

Table S17 PGAP annotations, those mapped to genes that were RBH in hot spring-derived strains and NH in non-thermal environment-derived strains, detected exclusively in *L*. sp. Akita

Table S18 PGAP annotations, those mapped to genes that were RBH in hot spring-derived strains and NH in non-thermal environment-derived strains, detected exclusively in *L*. sp. Seranma-*L*. sp. JSC-1-*L*. sp. O-77 lineage

Table S19 Results of Kruskal-Wallis test and glm test between read abundances of *B. buergeri* of 26 °C, *B. buergeri* of 28 °C, *B. buergeri* of 37 °C, and *B. japonica* of 40 °C

Table S20 Results of Welch's t-test and Wilcoxon test between read abundances of *B*. *buergeri* and *B*. *japonica*

Table S21 Results of Welch's t-test and Wilcoxon test between read abundances of *B. buergeri* of 26 °C and *B. buergeri* of 28 °C

Table S22 Results of Welch's t-test and Wilcoxon test between read abundances of *B. buergeri* of 26 °C and *B. buergeri* of 37 °C

Table S23 Results of Welch's t-test and Wilcoxon test between read abundances of *B. buergeri* of 28 °C and *B. buergeri* of 37 °C

Table S24 BLAST search results of *Leptolyngbya*-assigned reads from tadpole intestinal samples against the 16S rRNA gene sequences of *Leptolyngbya*-related strains used in this study.
